# Communicative and psycholinguistic abilities in children with phenylketonuria and congenital hypothyroidism

**DOI:** 10.1590/S1678-77572009000700012

**Published:** 2009

**Authors:** Mariana Germano GEJÃO, Amanda Tragueta FERREIRA, Greyce Kelly SILVA, Fernanda da Luz ANASTÁCIO-PESSAN, Dionísia Aparecida Cusin LAMÔNICA

**Affiliations:** 1 MSc, Speech Language Pathologist; Department of Speech-Language Pathology and Audiology, Bauru School of Dentistry, University of São Paulo, Bauru, SP, Brazil.; 2 Speech Language Pathologist, Graduate student; Department of Speech-Language Pathology and Audiology, Bauru School of Dentistry, University of São Paulo, Bauru, SP, Brazil.; 3 MSc; Speech Language Pathologist; Municipal Public Service, Uru, SP, Brazil.; 4 Speech Language Pathologist, Graduate student; Neonatal Screening Laboratory of the Association of Parents and Friends of Special Needs Individuals (APAE), Bauru, SP, Brazil.; 5 PhD, Associate Professor; Department of Speech-Language Pathology and Audiology, Bauru School of Dentistry, University of São Paulo, Bauru, SP, Brazil.

**Keywords:** Phenylketonurias, Congenital hypothyroidism, Communication, Child

## Abstract

The Neonatal Screening for Inborn Errors of Metabolism of the Association of Parents and Friends of Special Needs Individuals (APAE) - Bauru, Brazil, was implanted and accredited by the Brazilian Ministry of Health in 1998. It covers about 286 cities of the Bauru region and 420 collection spots. Their activities include screening, diagnosis, treatment and assistance to congenital hypothyroidism (CH) and phenylketonuria (PKU), among others. In 2005, a partnership was established with the Department of Speech-Language Pathology and Audiology, Bauru School of Dentistry, University of São Paulo, Bauru, seeking to characterize and to follow, by means of research studies, the development of the communicative abilities of children with CH and PKU. Objective: The aim of this study was to describe communicative and psycholinguistic abilities in children with CH and PKU. Materials and Methods: Sixty-eight children (25 children aged 1 to 120 months with PKU and 43 children aged 1 to 60 months with CH) participated in the study. The handbooks were analyzed and different instruments were applied (Observation of Communication Behavior, Early Language Milestone Scale, Peabody Picture Vocabulary Test, Gesell & Amatruda's Behavioral Development Scale, Portage Operation Inventory, Language Development Evaluation Scale, Denver Developmental Screening Test, ABFW Child Language Test-phonology and Illinois Test of Psycholinguistic Abilities), according to the children's age group and developmental level. Results: It was observed that the children with PKU and CH at risk for alterations in their developmental abilities (motor, cognitive, linguistic, adaptive and personal-social), mainly in the first years of life. Alterations in the psycholinguistic abilities were also found, mainly after the preschool age. Attention deficits, language and cognitive alterations were more often observed in children with CH, while attention deficits with hyperactivity and alterations in the personal-social, language and motor adaptive abilities were more frequent in children with PKU. Conclusion: CH and PKU can cause communicative and psycholinguistic alterations that compromise the communication and affect the social integration and learning of these individuals, proving the need of having these abilities assisted by a speech and language pathologist.

## INTRODUCTION

Neonatal screening program (NSP) is the popular name attributed to the Neonatal Screening for Inborn Errors of Metabolism, which has the objective of detecting early congenital hypothyroidism (CH) and phenylketonuria (PKU), among other alterations that can cause intellectual deficiency^7^.

The NSP of the Association of Parents and Friends of Special Needs Individuals/Bauru (APAE-Bauru) was implanted and accredited by Brazilian Ministry of Health in 1998. It covers approximately 286 cities of the Bauru area, totalizing 420 collection spots. Their activities include screening, diagnosis and long-term assistance for CH and PKU. The multidisciplinary team for the assistance to the individuals is composed by a pediatrician, an endocrinologist, a nutritionist, a psychologist, a neurologist, a social assistant, a speech language pathologist and a biochemist. This is a pioneering work in this area because the speech language pathologist is not included in the team of professionals proposed by the Ministry Health. However, studies have shown communicative, psycholinguistic, cognitive, motor and personal-social developmental alterations, even in children with early beginning of treatment^3^^,^^5^^,^^10^^,^^15^^,^^18^^,^^22^^,^^25^^,^^27^^–^^29^.

CH is a systemic metabolic disturbance caused by insufficient production of thyroid hormones due to thyroid gland malformation or alterations in hormonal biosyntheses^20^^,^^21^. These hormones have great influence in the central nervous system (CNS) development because the vascularization, myelinization, dendritic trees, synapse formation, neuronal migration and genes expression depend on them^3^^,^^22^^–^^25^.

PKU is an autosomal recessive disorder, resulting from the mutation of the gene located in chromosome 12q22-24.1^6^. PKU is caused by the lack of an enzyme known as phenylalanine hydroxylase. This enzyme is responsible for converting the amino acid phenylalanine to a second amino acid, tyrosine, in the liver^27^. The alterations found in the brain tissue of individuals with PKU are nonspecific and of diffuse nature, and might compromise the CNS maturation, produce flaws in the myelinization, and interfere in the biochemical processes that affect some neurotransmitters.

The objective of this study was to describe communicative and psycholinguistic abilities in children with CH and PKU.

## MATERIAL AND METHODS

After approval by the Research Ethics Committee (Protocol #14/2005) of the Bauru School of Dentistry, University of São Paulo, the parents were asked to sign an informed consent form according to 196/96 Resolution. The study was developed in partnership with one of the six São Paulo State NSP centers, accredited by the Ministry of Health.

The criteria for the participants’ eligibility were: having early diagnosis^7^ for CH (TSH above 10 μIU/mL and T4 free below 0.75 mg/dL) or for PKU^7^^,^^9^ (PHE levels above 4 mg/dL); attending periodic follow up according to the national guidelines^1^; not presenting other congenital or acquired alterations apart from those of CH and/ or PKU; being aged less than 120 months for PKU and 60 months for CH.

Sixty-eight individuals of both genders aged 1 to 120 months were enrolled, being 25 children in the PKU group (PKUG) and 43 children in the CH group (CHG). The clinical history was collected by review of the medical records. The following evaluation instruments were used according to the age group:

*Early Language Milestone Scale^11^ (ELMS): to evaluate the visual, receptive auditory and expressive auditory functions of children under 36 months of age.

*Peabody Picture Vocabulary Test^12^ (PPVT): to evaluate the receptive vocabulary of children over 36 months of age.

*Gesell and Amatruda's Behavioral Development Scale^17^ (GABDS): to evaluate the adaptive motor, fine motor, gross motor, language and personal-social behavior of children under 72 months of age.

*Portage Operation Inventory^30^ (POI): to evaluate the motor, language, cognition, socialization and self-car behavior of children under 72 months of age.

*Language Development Evaluation Scale^21^ (LDES): to evaluate the receptive and expressive language development of children under 83 months of age.

*Denver Developmental Screening Test^13^ (DDST-II): to evaluate the fine motor, adaptive motor, gross motor, language and personal-social behavior of children under 72 months of age.

*ABFW Child Language Test-phonology^2^ – (ABFW): to evaluate the phonology of children over 36 months of age.

*Illinois Test of Psycholinguistic Abilities^4^ (ITPA): to evaluate the 12 psycholinguistic abilities subtests of children over 36 months of age (auditory reception, visual reception, auditory association, visual association, auditory memory, visual memory, auditory closure, visual closure, grammatical closure, verbal expression, manual expression, sounds combination).

*Observation of Communication Behavior (OCB): to evaluate the communicative function, comprehension, dialogue maintenance, symbolic play and time attention in all children of the study.

Descriptive statistical analysis was used in the results obtained for the CHG and PKUG. The Spearman's correlation test was used to determine the correlations among the instruments employed in the study. A significance level of 5% was set for all analyses.

## RESULTS

**Figure 1:**
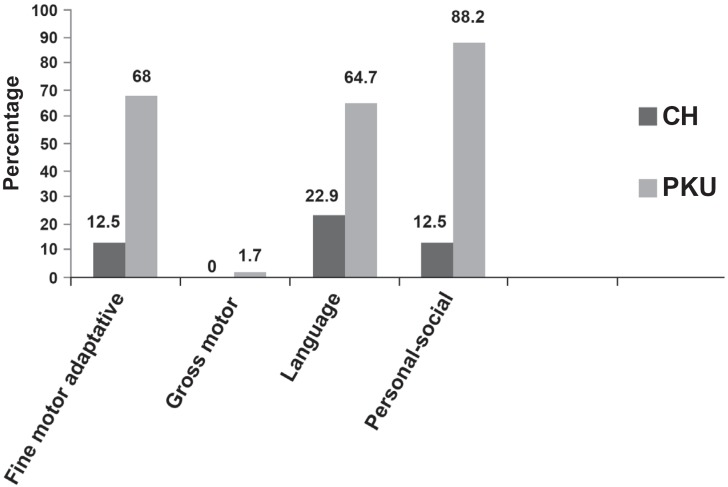
Percentage of children with alterations in the abilities of the DDST-II

Figure 1 presents, in percentage, the results of the alterations in the abilities evaluated in the DDST-II of 43 children with CH and 17 children with PKU.

**Figure 2:**
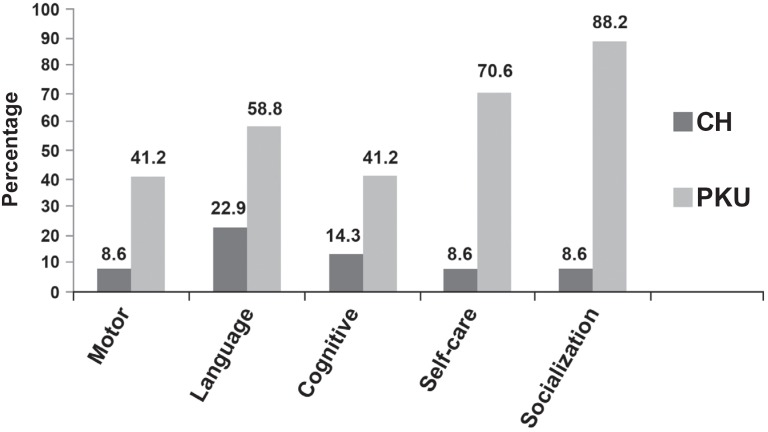
Percentage of children with alterations in the abilities of the POI

Figure 2 presents, in percentage, the results of the alterations in the abilities evaluated in the POI of 43 children with CH and 17 children with PKU.

**Figure 3:**
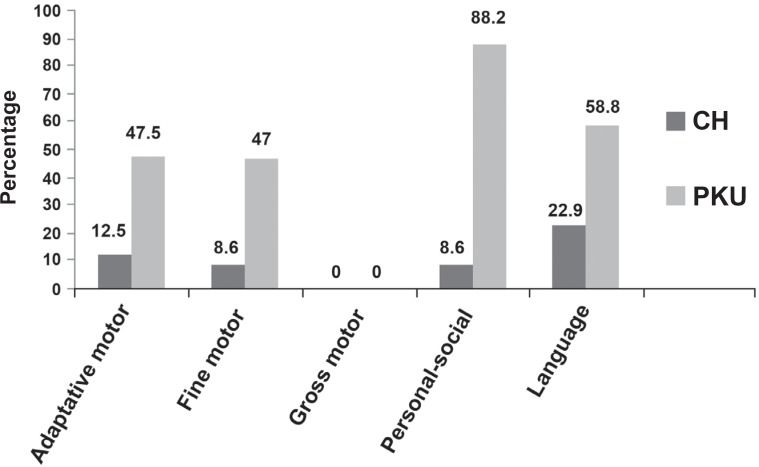
Percentage of children with alterations in the abilities of the GABDS

Figure 3 presents, in percentage, the results of the alterations in the abilities evaluated in the GABDS of 43 children with CH and 17 children with PKU.

**Figure 4:**
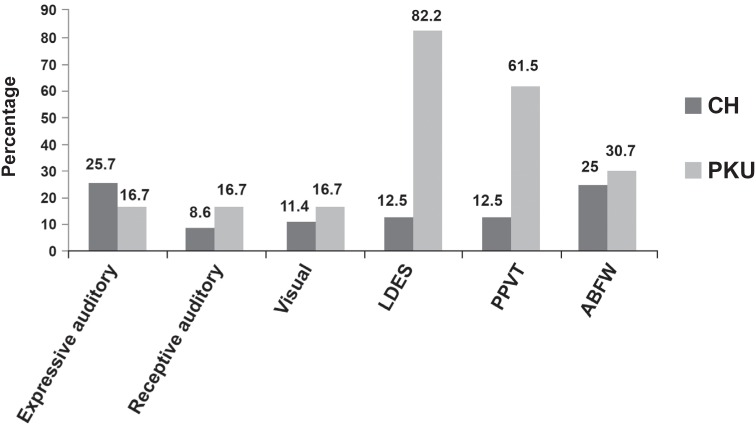
Percentage of children with alterations in the abilities of the ELMS, LDES, PPVT and ABFW

Figure 4 presents, in percentage, the results of the alterations in the abilities evaluated in the ELMS (expressive auditory, receptive auditory and visual) of 35 children with CH and 12 children with PKU; in the LDES of 43 children with CH and 17 with PKU; in the PPVT of 8 children with CH and 13 with PKU; and in the ABFW of 8 children with CH and 13 with PKU.

**Figure 5:**
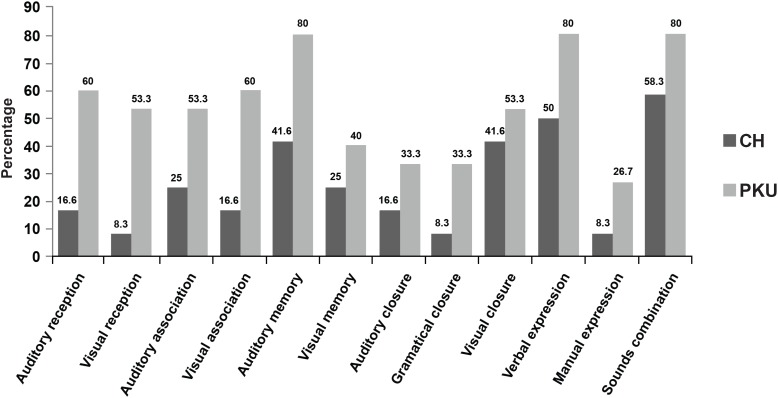
Percentage of children with alterations in the abilities of the ITPA

Figure 5 presents, in percentage, the results of the alterations in the abilities evaluated in the ITPA of 12 children with CH and 15 with PKU.

In the OCB, the CHG and PKUG presented verbal order comprehension, protesting, requesting, offering and informing functions, and symbolic play allowing dialogical activities. In both groups, the children demonstrated difficulty in attention time maintenance (39.5% for CHG and 64% for PKUG). The PKUG also presented hyperactivity (32%). These data were confirmed by the review of the clinical history.

There was statistically significant correlation among the evaluation instruments, which means that the instruments used in the study had similar capacity to evaluate the same ability.

## DISCUSSION

Analyzing the Figures 1 to 3, the CHG presented worse performance in the language area and PKUG in the personal-social area, followed by the language and motor fine-adaptive areas. The literature refers that delays in the oral language acquisition are frequent in CH^3^^,^^15^^,^^24^ and that individuals with PKU are of risk for alterations in their personal-social^5^, psycholinguists^10^^,^^22^^,^^29^ and fine motor coordination^14^ abilities. Few works focused the language specifically^28^. The language is a superior mental function, which depends on the CNS integrity, sensorial, perceptual, cognitive and maturational processes, and the environment influence^15^. The development field influence on the child's general performance has been emphasized. In other words, the language is the mediator for the child planing actions and interacting with the social environment. Therefore, alterations in the receptive or expressive language performance affect other development fields, mainly the adaptive and personal-social skills, interfere in the language development. As far as the expressive aspects are concerned (ELMS, LDES, ABFW, ITPA-verbal expression), CHG and PKUG did not present significant alterations for the phonology, but for the language use.

It has been reported that the longer the period of insufficient thyroid hormone production, the more severe and more extensive the cerebral damages because there will be alterations in the neuronal connections reducing the stimulus transmission capacity^3^^,^^18^^,^^20^^,^^24^. Individuals who cannot maintain the recommended phenylalanine levels can present alterations in the chemical mechanisms of the solid neurotransmitters with pre-frontal and/or left hemisphere dysfunction^29^, which affects the general learning.

Comparing the performance of CHG and PKUG in PPVT and LDES (Figure 4), PKUG presented more extensive damages. These data were confirmed by the Spearman's correlation test, proving that children that failed in one of the tests, in a given ability, also failed in the correlated abilities in another test. It means that these instruments were sensitive to detect the appraised ability profile and to confirm the tested hypothesis. It is emphasized that CH treatment is accomplished by hormonal replacement^7^^,^^19^^,^^23^^,^^24^, while PKU treatment involves a phenylalanine-poor diet^3^^,^^5^^–^^7^^,^^10^^,^^16^^,^^27^, making PKU control much more complex, especially in older children.

In ITPA, the most extensive damages were observed in CHG compared to PKUG. In OCB, difficulty was verified in the attention time maintenance for CHG and PKUG, which also presented hyperactivity. It is inferred that the results in ITPA suffered influence of the difficulty in the attention time maintenance. These symptoms have been extensively discussed in the literature^1^^,^^3^^,^^8^^,^^14^^,^^18^^,^^25^^–^^26^. In other words, attention deficit disorders, impulsiveness and hyperactivity are observed in CH^3^^,^^20^^,^^25^ and PKU^1^^,^^14^^,^^22^^,^^28^, and brings interference in attention, concentration, memory, analysis, synthesis abilities and executive functions performance, which depends on the appropriate CNS operation.

In this study, measurement of TSH, T4 and phenylalanine levels was done periodically during the treatment and the values were not correlated. Neurological imaging exams were also performed during the course of the treatment. These analyses are objective of future studies.

With the exposed so far, it is inferred that the treatment accomplished by the SNP in children with CHG and PKUG was efficient for the prevention intellectual deficit, but not for prevention of alterations in the communicative and psycholinguistic abilities that compromise the communication and interfere in the social integration and learning, indicating the need of assistance for these abilities by a speech language pathologist.

## CONCLUSION

In the studied population, children with CH and PKU presented alterations in their developmental abilities (linguistics, personal-social and fine- adaptive motor), mainly in the first years of life. There were significant alterations mainly in the visual and auditory psycholinguistic abilities of school children. The importance of having these children assisted is emphasized, seeking the prevention of communicative and psycholinguistic alterations as well as the increase of their social integration in the family and school environment.
